# Tracking tumor evolution via prostate-specific antigen: an individual post-operative study

**DOI:** 10.1186/1742-4682-7-30

**Published:** 2010-07-30

**Authors:** Mehmet Erbudak, Ayşe Erzan

**Affiliations:** 1 Laboratory for Solid State Physics, ETH Zurich, CH-8093 Zurich, Switzerland; 2 Department of Physics, Faculty of Letters and Sciences, Istanbul Technical University, Maslak, 34469 Istanbul, Turkey

## Abstract

**Background:**

The progress of the prostate-specific antigen (PSA) level after radical prostatectomy is observed for a patient in order to extract information about the mode of tumor cell growth. Although PSA values are determined routinely to find the doubling time of the prostate marker, to our knowledge, this analysis is the first in the literature.

**Results:**

The prostate tumor marker values were determined regularly after the surgery and plotted on a logarithmic scale against time. An initial rapid-growth mode changed to a slower power-law regime within two years of surgery. Our analysis associates this observation with a transition in the growth mode from unrestricted growth of dispersed cells to their clumping into macroscopic structures.

**Conclusions:**

Such studies may help determine the appropriate time window for postoperative therapies in order to increase the life expectancy of the patient.

## Background

Cancer of the prostate gland is one of the most frequently diagnosed male illnesses and may lead to death of the patient. The carcinoma is routinely detected by a straight-forward blood test that measures a glycoprotein called prostate-specific antigen (PSA). At an early stage of cancer growth with a localized tumor, radical removal of the prostate gland has proved to be the optimum treatment. If the PSA value rises after radical prostatectomy, different alternatives for treatment are currently under debate. The doubling time (DT) of the PSA value is accepted as a strong prognostic factor for the risk of cancer death. In a group of 379 patients, almost no prostate cancer deaths were recorded within approximately 4 years of prostate removal for 3 < DT < 8.9 months, while some patients with DT < 3 months died within 1.5 years [1]. It is therefore reasonable to infer that findings during the last few years based on long-term statistics suggest a longer life expectancy for patients with postoperative radiotherapy that follows (within 6 months of) radical prostate surgery [2]. Transdermal radiotherapy is commonly applied after the PSA level reaches a threshold value. However, a wait-and-watch method may cost valuable time and the relevant moment for action may be missed regardless of how low the threshold value is set. Many authors have already suggested [3-5] that the entire course of tumor growth offers important information regarding the clinical strategies to be followed.

The purpose of this work is threefold. (a) To indicate the possibility of detecting fast (exponential) growth of the PSA score well before an arbitrary threshold value is reached, thus gaining time for deciding the therapies to be followed. In principle, this strategy is analogous to determining the DT. (b) To analyze PSA data in a way that reveals a sharp crossover from exponential to power-law growth. (c) To propose a simple model to explain the crossover to slower (power-law) growth.

The second, slower growth regime, we argue, arises because of the coalescence or "condensation" [6] of freely-dividing cancerous cells to form one or more compact tumors, with growth essentially confined to the edges or the surface [4,5,7]. It has been pointed out that at this stage *(i) *"sensitivity to anti-metabolic drugs decreases, ... (since the fraction of) tumor cells that are in the cell division cycle decreases" [3] and *(ii) *angiogenesis is expected to start [5].

## Results

### Case presentation

**Figure 1:**
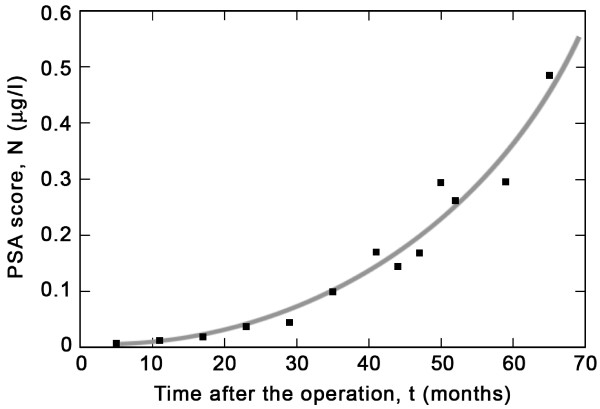
**Linear plot of PSA score vs. time**. PSA values in μg/l are displayed as a function of measurement time in months after the surgery.

After a radical prostatovesiculectomy (pT2c N0 M0 G2, Gleason 3+4 = 7) applied to one of us (ME), PSA values were determined with state-of-the-art precision using constant laboratory conditions (Viollier, Brunngasse 6, CH-8401 Winterthur) at time intervals of initially 6 months (see Table 1). The error in time measure was ± 0.5, while each PSA value was determined with an uncertainty of ± 0.002 μg/l. In Figure 1 we plot the values listed in the table as a function of time *t *in months after the surgery. The graph has a characteristic "U" shape, i.e., a shallow increase during the first two years and a steep rise in the last two.

**Table 1:** Measured PSA scores

Date of the PSA test	Time after the operation (months)	PSA score (μg/l)
Aug. 2003	5	0.006
Feb. 2004	11	0.012
Aug. 2004	17	0.019
Feb. 2005	23	0.037
Aug. 2005	29	0.044
Feb. 2006	35	0.099
Aug. 2006	41	0.170
Nov. 2006	44	0.144
Feb. 2007	47	0.168
May 2007	50	0.294
Jul. 2007	52	0.262
Feb. 2008	59	0.295
Aug. 2008	65	0.485

The PSA values and the dates of measurement after the operation in March 2003 as well as the time elapsed thereafter.

**Figure 2:**
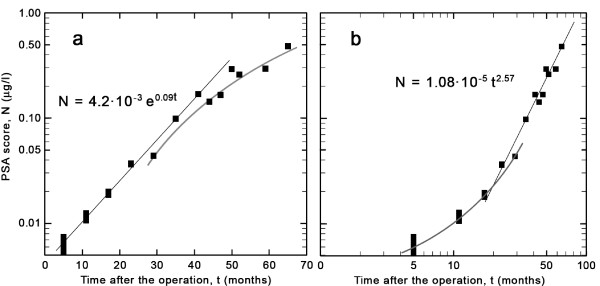
**Logarithmic plot of PSA score vs. time**. PSA values plotted on a logarithmic scale against (a) linear time and (b) time on a logarithmic scale. Note that the straight line fit in panel (a) signals exponential growth, while that in panel (b) indicates "power-law" growth (see text). The vertical size of the first four points indicates the estimated error, while in the subsequent points the error bars are smaller than the size of the plotted points.

In order to determine the kinetics of cell growth underlying the PSA progression, we present the same PSA values as a function of time in Figure 2a, but plotted on a semi-logarithmic scale. From the time of surgery until about 30 months thereafter we observe a clear linear increase. Analytically, this corresponds to "exponential growth", with the functional form ~exp(*pt*), where *p *is a constant rate of cell division. This can be determined at an early stage, before an arbitrary threshold value is attained.

Later values are noisier and remain below the straight line, implying a different, slower growth law. In Figure 2b we examine the departure from exponential behavior by displaying the PSA values vs. time on a log-log plot. Here a straight line signifies a power law (with the functional form ~*t*^*u*^). There is sharp crossover from exponential to power-law behavior at about two years after the surgery, rather than a gradual slowing down. From this point on, up to the last measured value, the PSA values grow as a power of the time elapsed after the crossover point.

### Growth modes

We assume that the PSA value is linearly proportional to the number *N *of carcinoma cells and that, initially, each cell freely divides at a constant rate *p *(probability per unit time). For such unrestricted growth, the number of cells, *N*, increases by *pN *per unit time on average, i.e.,(1)

At any time *t *from the start of the growth process, *N *is found to be(2)

where *N*_0 _is the number at *t *= 0. The growth rate *p *is the only relevant parameter that has to be experimentally determined. In this type of growth, the birth of a new cell has no effect on that of subsequent cells.

As the number of malignant cells grows within the tissue, there must be a spontaneous formation of macroscopic clusters of cells (i.e., tumors) that mop up almost all the microscopic clusters, at a rather sharp transition point, the so-called percolation threshold [8]. We identify this threshold with the crossover observed in Figure 2b. Once clusters are formed, growth is confined to the surface of the tumor [5] and the kinetic equations should only involve the number *N*_*s *_of actively dividing cells in the surface layer.

The number of cells in the tumor is roughly *N *~ *R*^*D *^where *R *is the average radius and *D *is the dimension of the cluster. The number in the surface layer will grow as *N*_*s *_~ *R*^*S*^, with *S *being the surface dimension [9]. In the most general case where *D *≤3, Equation 1 is replaced by(3)

where *k*_*1 *_and *k*_*2 *_are constant geometrical factors, leading to(4)

Here , with *N*_*0 *_having the same meaning as in Equation 1, and *c*_*2 *_= *pk*_*2*_. Irrespective of the exact value of the exponent, for a sufficiently small *c*_*1*_, Equation 3 predicts power-law growth. For spheroidal tumors [7] with growth confined to the surface region, *u *= 3. The parameter *u *is related to the compactness or looseness of the clumps of malignant cells and has to be determined experimentally.

### Data analysis

Figure 2a confirms exponential growth of the initial PSA scores with a rate of *p *= 0.090 ± 0.004 per month. Thus, the DT is about 8 months [DT in months is given by (ln 2)/p], or the PSA score increases by more than a factor of three within a year. In current practice, each datum point at a particular time is used by the physician to assess the patient's health condition and to decide upon further action. Although the absolute PSA values are much lower than the widely accepted threshold values for the recurrence of prostate cancer, the growth rate is alarmingly fast. Yet after about three years, the growth rate has slowed down and is seen to deviate from exponential behavior. Nevertheless, any arbitrarily set threshold will eventually be attained.

The log-log plot of the PSA values vs. time in Figure 2b shows a crossover from an exponential to a power law. The value of the exponent is *u *= 2.57 ± 0.07, very close to what would be predicted for percolation clusters [8,9] with growth confined to an outer shell.

The values for the coefficient *p *in the exponential form exp(*pt*) and the exponent *u *in the form *t*^*u *^are found from least squares fits to the linear parts of the plot in the respective cases. It should be emphesized that the main import of the paper is not the precise numerical values of *p *or *u*, although with a linear fit to the straight lines in the two plots these can be determined to an accuracy of two (one) significant digits (digit), respectively, with Pearson's correlation coefficients of *r*^2 ^= 0.98 and 0.96. The point is that there is an unambiguous crossover, from a characteristically exponential to a characteristically power-law behavior.

## Discussion

The "Gompertzian" [10] growth curve widely accepted as a "universal law" [3-7,11-14] for the growth of diverse populations including tumors and cell cultures [13] exhibits initial exponential growth, gradually slowing down and finally saturating (*in vitro*) to a constant value. We find that the data reported in Table 1 are also fitted reasonably well by a Gompertzian (see Figure three of Ref [14]). It would be worthwhile to re-plot the data traditionally analyzed within this gradualist picture on a log-log scale, and see whether the same sharp crossover behavior is actually hiding there as well.

Once the switch to power-law growth occurs, signalling discrete, compact tumors, we can estimate their size assuming that the PSA value is linearly proportional to the total number of malignant cells and knowing the PSA value and tumor size obtained from magnetic-resonance imaging (MRI) at the time of the operation.

In the particular case under study, the gland, prior to its removal, had a diameter of less than 40 mm (Huber D: *MRI Report*. Zurich: Klinik Hirslanden; 2002) and the PSA value was 8.5 μg/l. The prostate was about 50% cancerous according to the post-operative biopsy, so we deduce that a PSA level of 0.485 μg/l, measured at *t *= 65, would correspond to a tumor (consisting only of cancerous cells) approximately 5 mm in diameter. If not one but two equally-sized compact clusters condense out of the scatter of individual cells, our naive calculation gives a diameter of approximately 4 mm for each of those tumors. Digital rectal examinations by three independent experts (Brodmann S; Riesterer O; Vollenweider P; 2008) as well as the MRI analysis (Hilfiker P: *Medical Report*. Zurich: MRI Bethanien; 2008) performed at *t *= 67 revealed two masses of about 4 mm in agreement with our prediction. Subsequently, the subject received 70 Gy of radiation therapy in the anastomosing region during weekdays of *t *= 68 and 69 in equal doses, with full bladder and an inflated (50 ml) rectal balloon. The volume was reduced after 46 Gy in order to spare the vicinity. Since the radiation therapy, the PSA values have remained around 0.16 μg/l.

## Conclusions

We find that the PSA values of this patient after surgery follow an initial exponential growth curve, with a sharp crossover to a slower power-law regime at around 20 months. We argue that this may be due to the clumping of individual cancerous cells into macroscopic structures with distinct surfaces, to which subsequent growth is confined.

In the present case, after the onset of the power-law growth regime, it was possible to detect the clusters of cells, i.e., the tumors, via standard imaging techniques, and verify that their sizes coincided with predictions from the model.

Radiation therapy is not routinely applied after the surgery. The predictive power of our simple analysis, however, makes it highly worthwhile to monitor the PSA scores closely during the so-called wait-and-watch period no matter how low their absolute value is, as has been done in the present case, in order not to miss the optimum time window for post-operative therapy to increase the life expectancy of the cancer patient.

The doubling time is a widely-used characteristic of cell growth and a constant value testifies to exponential growth. The novel aspect of our analysis deals with the deviation from exponential behavior, which transforms into a subsequent power-law regime. Though mathematically convincing, this non-exponential late-stage growth behavior observed for one particular patient may not represent a universal phenomenon. However, monitoring the growth of the PSA value as a function of time may provide the opportunity for observing such a crossover, as for this patient. If such a crossover indeed occurs, it may indicate, as explained above, the formation of macroscopic masses within the tissue, and we suggest that this may be taken as a clue for deciding upon post-operative treatment.

## Consent

Written informed consent for publication was obtained from the patient, who is one of the authors (ME), for publication of this case report and accompanying images. A copy of the written consent is available for review by the Editor-in-Chief of this journal.

## Competing interests

The authors declare that they have no competing interests.

## Authors' contributions

The authors contributed equally to this work, and read and approved the final manuscript.

## References

[B1] Freedland SJ Humphreys EB Mangold LA Eisenberger M Dorey FJ Walsh PC Partin AW Death in patients with recurrent prostate cancer after radical prostatectomy: Prostate-specific antigen doubling time subgroups and their associated contributions to all-cause mortality J Clin Oncol 2007 25 1765 71 10.1200/JCO.2006.08.0572 17470867

[B2] Bolla M van Poppel H Collette L van Cangh P Vekemans K Da Pozzo L de Reijke TM Verbaeys A Bosset J-F van Velthoven R Maréchal J-M Scalliet P Haustermans K Piérart M Postoperative radiotherapy after radical prostatectomy: a randomised controlled trial Lancet 2005 366 572 578 10.1016/S0140-6736(05)67101-2 16099293

[B3] Schabel FM Jr The use of tumor growth kinetics in planning "curative" chemotherapy of advanced solid tumors Cancer Res 1969 29 2384 2389 5369685

[B4] Brú A Albertos S Subiza JL Garcia-Asenjo JL Brú I The universal dynamics of tumor growth Biophys J 2003 85 2948 2961 10.1016/S0006-3495(03)74715-8 14581197 PMC1303573

[B5] Kohandel M Kardar M Milosevic M Sivaloganathan S Dynamics of tumor growth and combination of anti-angiogenic and cytotoxic therapies Phys Med Biol 2007 52 3665 3677 10.1088/0031-9155/52/13/001 17664569

[B6] Torkington P Kinetics of deterrence of Gompertzian growth Bull Math Biol 1983 45 21 31 6850158 10.1007/BF02459384

[B7] Delsanto PP Guiot C Degiorgis PG Condat CA Mansury Y Deisboek TS Growth model for multicellular tumor spheroids App Phys Lett 2004 85 4225 4227 10.1063/1.1812842

[B8] Stauffer D Aharony A Introduction to Percolation Theory 1992 London: Taylor and Francis

[B9] Mandelbrot BB The Fractal Geometry of Nature 1983 New York: Macmillan

[B10] Gompertz B On the nature of the function expressive of the law of human mortality, and on a new mode of determining the value of life contingencies Phil Trans R Soc 1825 115 513 583 10.1098/rstl.1825.0026 PMC4360127 25750242

[B11] DeWys WD Studies correlating the growth rate of a tumor and its metastases and providing evidence for tumor-related systemic growth-related factors Cancer Res 1972 32 374 379 4258017

[B12] Iwata K Kawasaki K Shigesada N A dynamical model for the growth and size distribution of multiple metastatic tumors J Theor Biol 2000 203 177 186 10.1006/jtbi.2000.1075 10704301

[B13] Norton L Simon R Brereton HD Bogden AE Predicting the course of Gompertzian growth Nature 1976 264 542 545 10.1038/264542a0 1004590

[B14] Guiot C Degiorgis PG Delsanto PP Gabriele P Deisboek TS Does tumor growth follow a "universal law"? J Theor Biol 2005 225 147 151 10.1016/S0022-5193(03)00221-2 14575649

